# The Coxsackievirus B 3C^pro^ Protease Cleaves MAVS and TRIF to Attenuate Host Type I Interferon and Apoptotic Signaling

**DOI:** 10.1371/journal.ppat.1001311

**Published:** 2011-03-10

**Authors:** Amitava Mukherjee, Stefanie A. Morosky, Elizabeth Delorme-Axford, Naomi Dybdahl-Sissoko, M. Steven Oberste, Tianyi Wang, Carolyn B. Coyne

**Affiliations:** 1 Department of Cell Biology and Physiology, University of Pittsburgh, Pittsburgh, Pennsylvania, United States of America; 2 Department of Microbiology and Molecular Genetics, University of Pittsburgh, Pittsburgh, Pennsylvania, United States of America; 3 Picornavirus Laboratory, Centers for Disease Control and Prevention, Atlanta, Georgia, United States of America; 4 Department of Infectious Diseases and Microbiology, University of Pittsburgh, Pittsburgh, Pennsylvania, United States of America; University of North Carolina at Chapel Hill, United States of America

## Abstract

The host innate immune response to viral infections often involves the activation of parallel pattern recognition receptor (PRR) pathways that converge on the induction of type I interferons (IFNs). Several viruses have evolved sophisticated mechanisms to attenuate antiviral host signaling by directly interfering with the activation and/or downstream signaling events associated with PRR signal propagation. Here we show that the 3C^pro^ cysteine protease of coxsackievirus B3 (CVB3) cleaves the innate immune adaptor molecules mitochondrial antiviral signaling protein (MAVS) and Toll/IL-1 receptor domain-containing adaptor inducing interferon-beta (TRIF) as a mechanism to escape host immunity. We found that MAVS and TRIF were cleaved in CVB3-infected cells in culture. CVB3-induced cleavage of MAVS and TRIF required the cysteine protease activity of 3C^pro^, occurred at specific sites and within specialized domains of each molecule, and inhibited both the type I IFN and apoptotic signaling downstream of these adaptors. 3C^pro^-mediated MAVS cleavage occurred within its proline-rich region, led to its relocalization from the mitochondrial membrane, and ablated its downstream signaling. We further show that 3C^pro^ cleaves both the N- and C-terminal domains of TRIF and localizes with TRIF to signalosome complexes within the cytoplasm. Taken together, these data show that CVB3 has evolved a mechanism to suppress host antiviral signal propagation by directly cleaving two key adaptor molecules associated with innate immune recognition.

## Author Summary

Mammalian cells utilize a variety of defenses to protect themselves from microbial pathogens. These defenses are initiated by families of receptors termed pattern recognition receptors (PRRs) and converge on the induction of molecules that function to suppress microbial infections. PRRs respond to essential components of microorganisms that are broadly expressed within classes of pathogens. The relative non-specificity of this detection thus allows for a rapid antimicrobial response to a variety of microorganisms. Coxsackievirus B3 (CVB3), a member of the enterovirus genus, is associated with a number of diverse syndromes including meningitis, febrile illness, diabetes, and is commonly associated with virus-induced heart disease in adults and children. Despite its significant impact on human health, there are no therapeutic interventions to treat CVB3 infections. Here we show that CVB3 has evolved an effective mechanism to suppress PRR signal propagation by utilizing a virally-encoded protein, termed 3C^pro^, to directly degrade molecules that function downstream of PRR signaling. By targeting these molecules, CVB3 can evade host detection and escape antiviral defenses normally induced by mammalian cells. These findings will lead to a better understanding of the mechanisms employed by CVB3 to suppress host antiviral signaling and could lead to the development of therapeutic interventions aimed at modulating CVB3 pathogenesis.

## Introduction

The innate immune system is the first line of defense against pathogen infiltration and is activated by the binding of conserved microbial ligands to pattern recognition receptors (PRRs). Activation of these receptors culminates in nuclear factor (NF)-κB and/or IFN regulatory factor (IRF)-mediated induction of type 1 interferons (IFN-α and -β), key components of antimicrobial host defenses.

PRRs, including Toll-like receptors (TLRs) and DExD/H box RNA helicases, signal through an assortment of downstream adaptor molecules to propagate innate immune signaling. TLRs signal through adaptor molecules such as myeloid differentiation factor 88 (MyD88), Toll/IL-1 receptor domain containing adaptor protein (TIRAP), Toll/IL-1 receptor domain containing adaptor inducing interferon-beta (TRIF), and TRIF-related adaptor molecule (TRAM) to activate cellular defenses [1]. These adaptors often display specificity with regard to the TLR family members with whom they interact with and from which they are activated. The specificity of TLR ectodomain-ligand recognition and concomitant specificity in the signaling networks that are engaged by this interaction provides an efficient strategy for microbial recognition. In contrast, activated DExD/H box RNA helicases, which include melanoma differentiation associated gene (MDA5) and retinoic acid induced gene-I (RIG-I), signal to a common downstream adaptor molecule, mitochondrial antiviral signaling [(MAVS), also known as VISA/IPS-1/Cardif] to activate NFκB and IRF3 [2], [3], [4]. MAVS is localized to the mitochondrial membrane and to peroxisomes via a C-terminal transmembrane domain, which is essential for innate immune signaling [5], [6]. PRR-associated adaptor molecules thus serve critical roles in the activation of cellular defenses associated with microbial recognition.

As host cells have developed highly specialized strategies for microbial detection and clearance, it is not surprising that many viruses have evolved strategies to counter these defenses in order to promote their replication and spread. In some cases, virally-encoded proteases directly target components of the innate immune system to abolish antiviral signaling via TLRs and/or DExD/H box helicases. Targeted proteolysis of adaptor molecules serves as a powerful means to eliminate antiviral signaling by suppressing common downstream targets of key innate immune signaling pathways. For example, MAVS is cleaved by the NS3/4A serine protease of hepatitis C virus (HCV) [7], the 3C^pro^ cysteine protease of hepatitis A virus (HAV) [8], the HCV-related GB virus B NS3/4A protease [9], and the 2A^pro^ and 3C^pro^ proteases of rhinovirus [10]. HCV also utilizes the same NS3/4A serine protease to cleave TRIF in order to silence TLR3-mediated signaling [11]. Thus, the targeting of MAVS and/or TRIF by virally-encoded proteases in order to suppress antiviral signaling is emerging as a common theme in the evasion of host defenses.

Enteroviruses, which belong to the *Picornaviridae* family, are small single-stranded RNA viruses that account for several million symptomatic infections in the United States each year. Coxsackievirus B3 (CVB3), a member of the *Enterovirus* genus, is associated with a number of diverse syndromes, including meningitis, febrile illness, and diabetes [12] and is an important causative agent of virus-induced heart disease in adults and children [13], [14], [15], [16]. The induction of type I IFN signaling is essential for the control of CVB3 infection, as evidenced by enhanced virus-induced lethality in type I IFN receptor (IFN-α∼β R) null mice [17] and increased susceptibility to CVB3 infection in IFNβ-deficient mice [18]. Both TLR3- and MDA5-mediated type I IFN signaling have been implicated in the response to CVB3 infections and mice deficient in either TRIF or MAVS show an enhanced susceptibility to viral infection [19], [20], [21].

In this study, we determined the potential mechanisms employed by CVB3 to antagonize type I IFN signaling. We found that infection of cells with CVB3 led to the cleavage of the adaptor molecules MAVS and TRIF. Both MAVS and TRIF were cleaved by the CVB3-encoded cysteine protease 3C^pro^, indicating that a single protease suppresses innate immune signaling through two powerful pathways. We found that 3C^pro^ cleaves specific residues within MAVS and TRIF that render these molecules deficient in type I IFN signaling and apoptotic signaling. Taken together, these data suggest that CVB3 has evolved a mechanism to cleave adaptor components of the innate immune system to escape host immunity.

## Results

### CVB3 infection does not induce IRF3 nuclear localization or significant type I IFN responses

**Figure 1:**
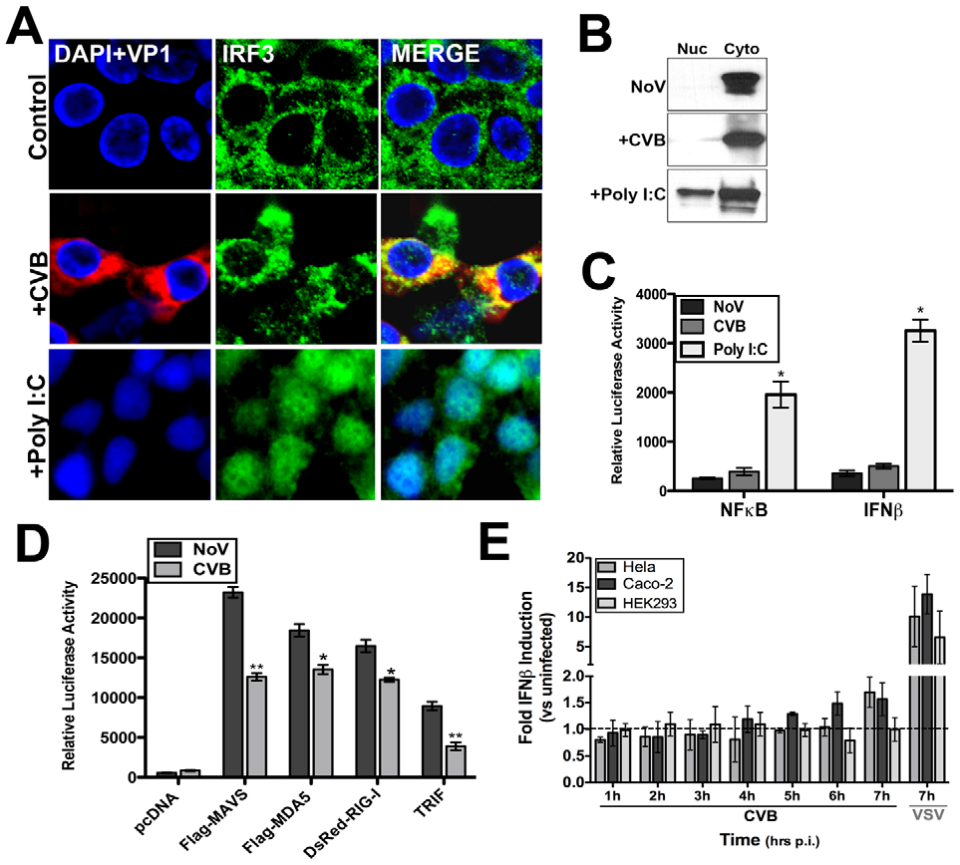
CVB3 infection does not induce significant type I IFN responses. HEK293 cells were infected with CVB3 (1 PFU/cell) for 8 hrs or treated with poly I:C conjugated to transfection reagent [poly I:C/LyoVec (100 ng/mL)] for 12 hrs and (**A**) fixed and stained for virus (VP1, red) and IRF3 (green) or (**B**) western blot analysis for IRF3 performed on nuclear and cytoplasmic fractions. (**C**) Luciferase assays (expressed in relative luciferase activity) from HEK293 cells transfected with NFκB and IFNβ promoted luciferase constructs and infected with CVB3 (8 hrs) or treated with 100 ng/mL poly I:C/LyoVec for 12 hrs. Data are shown as mean ± standard deviation. Asterisks indicate p-values of ≤0.05. (**D**) Luciferase assay (expressed in relative luciferase activity) from HEK293 cells transfected with the indicated constructs and IFNβ promoted luciferase constructs for 24 hrs and then infected with CVB3 (1 PFU/cell) for 14 hrs. (**E**), IFNβ production as measured by ELISA from HEK293, HeLa, or Caco-2 cell culture supernatants infected with either CVB3 (3PFU/cell) or VSV (5PFU/cell) for the indicated times. Data are shown as the fold IFNβ induction compared to no virus (NoV) controls. Data in (D) and (E) shown as mean ± standard deviation. Asterisks indicate p-values ≤0.05.

The induction of type I IFNs is the earliest cellular immune response initiated to combat viral infections and is coordinated by the activation of transcription factors such as interferon regulatory factor (IRF)-3, IRF7, and NFκB downstream of PRR activation. We found that CVB3 infection of HEK293 cells led to only a modest induction of IRF3 activation as assessed by immunofluorescence microscopy for nuclear translocation (Figure 1A), western blot analysis of nuclear extracts (Figure 1B), and luciferase activity assays for IFNβ (Figure 1C). In contrast, transfection of cells with poly I:C induced pronounced IRF3 activation (Figure 1A–C). We also observed little activation of NFκB signaling in response to CVB3 infection as determined by luciferase activation assay (Figure 1C).

Because CVB3 did not elicit a pronounced translocation of IRF3 into the nucleus during infection of HEK293 cells, we investigated the role of several PRRs in mediating CVB3 recognition–TLR3, RIG-I, and MDA5. Both MDA5 [22] and TLR3 [19] have been proposed to act as sensors for CVB3 infection. Although infection of cells with CVB3 is sensitive to IFNβ (Supplemental Figure S1A), we observed less enhancement of IFNβ promoter activity as assessed by luciferase activation in CVB3-infected HEK293 cells overexpressing MAVS, MDA5, RIG-I, and TLR3/TRIF than in uninfected controls (Figure 1D). Instead, we observed the partial ablation of IFNβ promoter activity in response to ectopic expression of MAVS, RIG-I, MDA5, and TLR3/TRIF in CVB3 infected cells (Figure 1D). We also found that CVB did not induce potent IFNβ production in HEK293, HeLa, or Caco-2 cells in comparison to VSV controls (Figure 1E).

### MAVS and TRIF are cleaved in CVB3-infected cells

**Figure 2:**
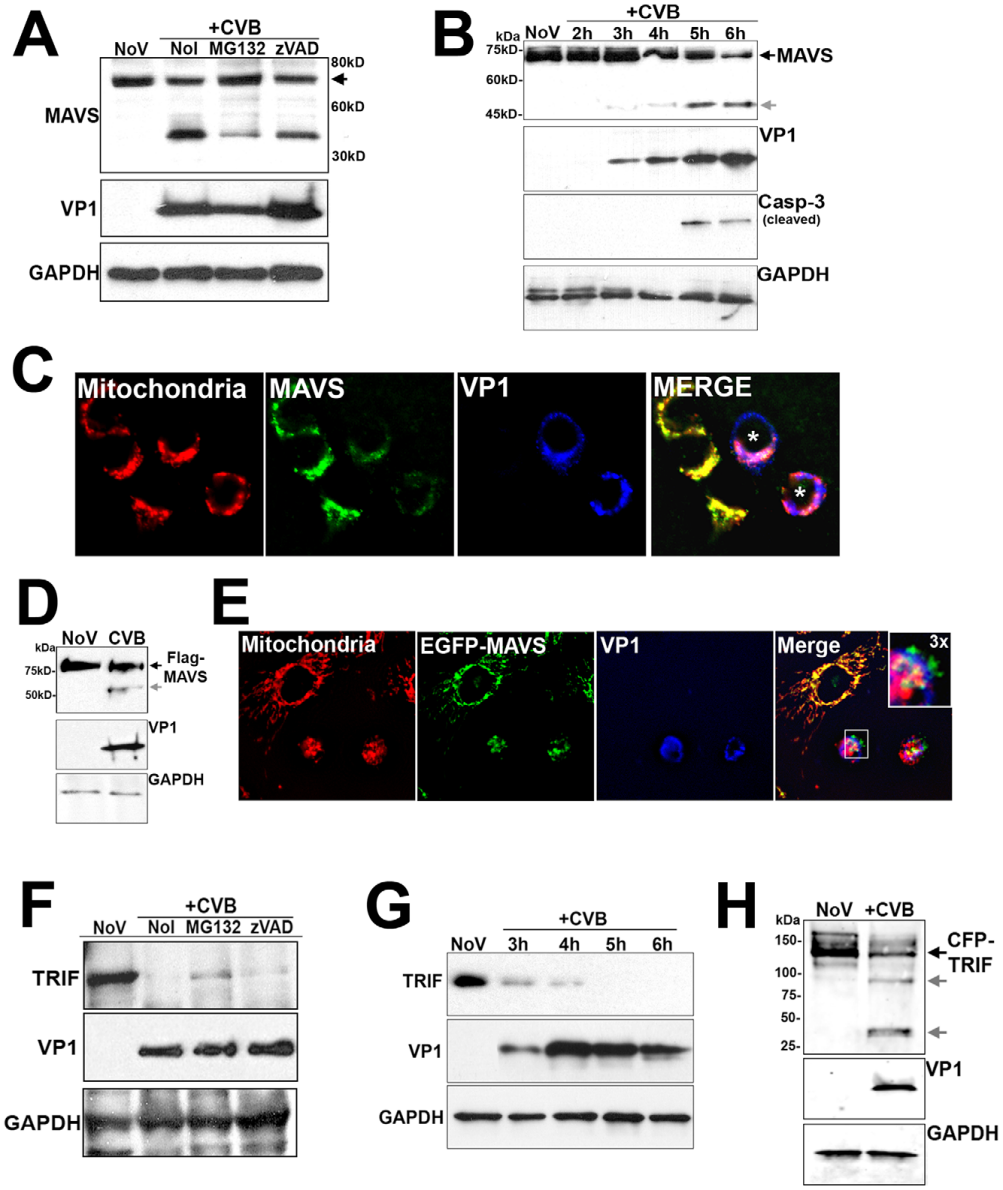
CVB3 infection induces MAVS and TRIF cleavage. (**A**) Western blot analysis for MAVS in HEK293 cells infected with CVB3 for 12 hrs in the absence (NoI) or presence of Z-VAD-FMK (zVAD) or MG132. (**B**) Time course of MAVS and caspase-3 cleavage in HEK293 cells infected with CVB3 for the indicated times. (**C**) HEK293 cells were infected with CVB3 for 8 hrs and fixed and stained for MAVS (green), mitochondria (red), and VP1 (blue). Asterisks denote infected cells expressing less MAVS than uninfected controls. (**D**, **E**) U2OS cells transfected with Flag-MAVS or EGFP-MAVS were infected with CVB3 (1 PFU/cell) for 7 hrs (D) or 12 hrs (E) and then lysed and immunoblotted with anti-Flag monoclonal antibody (D) or fixed and stained for mitochondria (red) and VP1 (blue) (E). In order to better visualize cleavage fragments in (D), CVB-infected cultures were transfected with 2 µg Flag-MAVS (in comparison to 1 µg in uninfected controls). (**F**) Western blot analysis for TRIF in HeLa cells infected with CVB3 for 8 hrs in the absence (NoI) or presence of z-VAD-FMK (zVAD) or MG132. (**G**) Time course of TRIF cleavage in HeLa cells infected with CVB3 (1 PFU/cell) for the indicated times. (**H**) Immunoblot analysis for overexpressed CFP-TRIF in HEK293 cells infected with CVB3 (1 PFU/cell) for 8 hrs. In (A), (D), and (H), grey arrows denote CVB3-induced cleavage fragments.

Because CVB3 infection was inefficient at inducing IRF3, we assessed the pattern of expression of MAVS in CVB3-infected HEK293 cells. By immunoblot analysis, we found that CVB3 infection induced the cleavage of MAVS (Figure 2A). Similar results were obtained in HeLa cells (Supplemental Figure S2A). This effect was specific for CVB3 as infection with VSV did not alter MAVS migration (Supplemental Figure S2B). In uninfected cells, full-length MAVS migrated as a single band of ∼75 kD. However, in cells infected with CVB3, there was a decrease in the expression level of full-length MAVS and the appearance of a distinct MAVS cleavage fragment migrating at ∼40–50 kD (Figure 2A). Because MAVS cleavage is induced in cells undergoing apoptosis [23], [24] and CVB3 is known to induce apoptosis in many cell types [25], [26], we investigated the role of apoptosis in CVB3-induced MAVS cleavage. We found that incubation of CVB3-infected HEK293 cells with the broad caspase inhibitor z-VAD-FMK and the proteosome inhibitor MG132 had little effect on CVB3-induced MAVS cleavage (Figure 2A). (The slight reduction in MAVS cleavage observed in the presence of MG132 is likely attributable to a reduction in replication in MG132-exposed cells, consistent with previously published results [27], [28]). The kinetics of MAVS cleavage was also not consistent with apoptosis: MAVS cleavage was evident by 3 hrs post-infection (p.i.) whereas apoptosis (as measured by caspase-3 cleavage) did not occur until 5–6 hrs p.i. (Figure 2B).

MAVS is localized to the mitochondrial membrane via a C-terminal transmembrane domain [5]. We found that CVB3 infection induced a pronounced decrease in MAVS mitochondrial localization as assessed by immunofluorescence microscopy with a mitochondrial marker (Figure 2C). We also found that the expression and mitochondrial localization of ectopically expressed MAVS was significantly reduced in CVB3-infected cells (Figure 2D, 2E). The appearance of cleavage fragments was evident in CVB3-infected cells overexpressing MAVS (Figure 2D). Moreover, we found that mutation of the caspase cleavage site of MAVS (D429E, [24]) had no effect on CVB3-induced MAVS cleavage (Supplemental Figure S2C), indicating a caspase-independent mechanism of action.

Another common pathway upstream of IRF3 activation is the engagement of TLR3 by viral dsRNA, which is produced as a replication intermediate during viral infection. As we observed cleavage of MAVS in CVB3-infected cells, we sought to determine if CVB3 might also target TRIF, the specific adaptor molecule downstream of TLR3, to repress IRF3 activation. Similar to our findings with MAVS, we found that TRIF expression was significantly reduced in HeLa cells infected with CVB3 (Figure 2F). Although TRIF can be cleaved during apoptosis [23], [24], we found that z-VAD-FMK and MG132 had little effect at antagonizing the CVB3-mediated reduction in TRIF expression (Figure 2F), consistent with our findings with MAVS (Figure 2A). The kinetics of TRIF cleavage also paralleled that of MAVS as we observed a marked reduction in TRIF levels by 3 hrs p.i. (Figure 2G), a time prior to the induction of caspase-3 cleavage (Figure 2B). Ectopically expressed CFP-fused TRIF was also significantly decreased in cells infected with CVB3 and coincided with the appearance of several cleavage fragments (Figure 2H).

We next investigated whether cleavage of MAVS and TRIF occurred in cells infected with other enteroviruses including echovirus 7 (E7) and enterovirus 71 (EV71). Infection of HeLa cells with both E7 and EV71 led to the significant reduction of MAVS and TRIF expression, which corresponded with the appearance of the newly replicated viral protein VP1 (Supplemental Figure S3A). However, in contrast to our findings with CVB3 (Figure 2A), we did not observe the appearance of any significant cleavage fragments in either E7 or EV71-infected cells. This may indicate that the cleavage fragments are short-lived in E7 or EV71-infected cells or that cleavage occurs at different residues within the molecule that alter antibody binding. These results may indicate that members of the enterovirus family target MAVS and TRIF to evade host immunity, but further studies are required to definitively show which members of the enterovirus family utilize this mechanism.

CVB3 infections are commonly associated with virus-induced heart disease in adults and children and have been detected in approximately 20-25% of patients with dilated cardiomyopathy and myocarditis [13], [14], [15], [16]. To determine whether MAVS and TRIF are degraded *in vivo*, mice were infected with CVB3 and the hearts of infected animals were probed for MAVS and TRIF. In contrast to uninfected controls, there was an almost complete absence of both MAVS and TRIF in murine hearts infected with CVB3 (Supplemental Figure S3B). These data indicate that the cleavage of MAVS and TRIF may also occur during CVB3 infection *in vivo*.

### CVB3 3C^pro^ cleaves MAVS and TRIF

**Figure 3:**
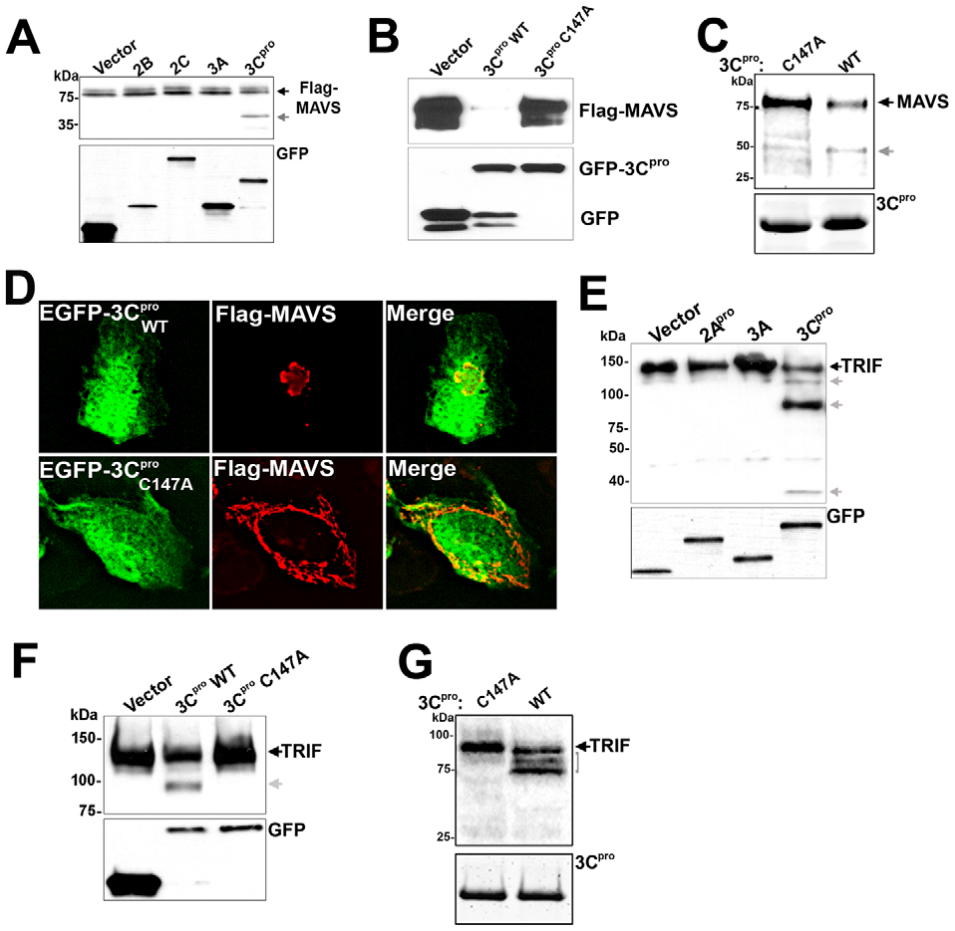
3C^pro^ Cleaves MAVS and TRIF. (**A**) Immunoblot analysis for Flag-MAVS (top) and GFP (bottom) in HEK293 cells co-transfected with EGFP-2B, 2C, 3A, or 3C^pro^ and Flag-MAVS. In order to better visualize cleavage fragments, EGFP-3C^pro^ expressing cells were transfected with 2 µg Flag-MAVS (in comparison to 1 µg with other constructs). (**B**) HEK293 cells transfected with Flag-MAVS and control (EGFP-C2), EGFP-3Cpro wild-type or C147A were lysed and subjected to immunoblotting for MAVS and GFP. (**C**) 3C^pro^ cleaves MAVS *in vitro*. Recombinant wild-type or C147A mutant SUMO-3C^pro^ (10 µg) was incubated with column purified Flag-MAVS (0.1 µg) for 8 hrs at 37°C, fractionated by SDS-PAGE, and immunoblotted with anti-Flag monoclonal antibody (top) or commassie stained (bottom). (**D**) U2OS cells were transfected with EGFP-3C^pro^ wild-type (WT) or the C147A mutant and Flag-MAVS and immunofluorescence microscopy performed for MAVS (in red). (**E**) Immunoblot analysis of HEK293 cells transfected with CFP-TRIF and either vector control, EGFP-2A^pro^, 3A EGFP-3C^pro^ and lysates immunoblotted for TRIF (top) or GFP (bottom). Grey arrows denote 3C^pro^-induced cleavage products. (**F**) Lysates of HEK293 cells transfected with CFP-TRIF and vector, wild-type (WT), or C147A mutant EGFP-3C^pro^ were immunoblotted with anti- TRIF antibody (top) or anti-GFP (bottom) antibodies. Grey arrow denotes 3C^pro^-induced cleavage product. (**G**) 3C^pro^ cleaves TRIF *in vitro*. Recombinant wild-type or C147A mutant SUMO-3C^pro^ (10 µg) was incubated with column purified Flag-TRIF (0.1 µg) for 12 hrs at 37°C, fractionated by SDS-PAGE, and immunoblotted with anti-Flag monoclonal antibody (top) or commassie stained (bottom).

Enteroviruses encode specific proteases that are required for the processing of viral proteins and the establishment of replication, but which also cleave a variety of host cell molecules [29]. Because we observed the cleavage of MAVS in CVB3-infected cells, we investigated whether virally-encoded proteases might mediate this effect. We cotransfected HEK293 cells with N-terminal Flag-MAVS and various CVB3 viral proteins fused to EGFP. Of these proteins, we found that expression of the protease 3C^pro^ was sufficient to induce a significant reduction in MAVS expression (Figure 3A). In fact, in order to observe significant levels of full-length Flag-MAVS (or cleavage fragments) in EGFP-3C^pro^ co-transfected cells, cells had to be transfected with twice as much Flag-MAVS as vector control or other CVB3 viral proteins. The apparent lack of cleavage products in cells overexpressing proteases is a phenomenon that has also been observed for HCV-mediated cleavage of TRIF [11] and likely reflects the high efficiency of cleavage (which may result from protease overexpression) and that cleavage fragments are unstable and/or short-lived. For our subsequent studies, we transfected cells with equivalent amounts of MAVS cDNA to compare the level of full-length MAVS in control versus 3C^pro^-transfected cells. The cleavage of MAVS required the cysteine protease activity of 3C^pro^, as cotransfection of a catalytically inactive N-terminal EGFP-tagged 3C^pro^ mutant (C147A) [30] had no effect on MAVS expression (Figure 3B). In some cases, significant levels of GFP signal alone can be detected in EGFP-3C^pro^ WT transfected cells which is likely indicative of 3C^pro^ cleaving itself from the N-terminal EGFP tag.

To confirm that 3C^pro^ was directly cleaving MAVS, we incubated recombinant wild-type or C147A mutant 3C^pro^ with Flag-MAVS purified by Flag column affinity purification from overexpressing HEK293 cells. Whereas incubation with wild-type 3C^pro^ induced the appearance of a MAVS cleavage fragment as determined by Flag immunoblotting, the C147A mutant did not induce the appearance of a MAVS cleavage product (Figure 3C). Moreover, whereas expression of wild-type EGFP-3C^pro^ induced the relocalization of MAVS as assessed by immunofluorescence microscopy, expression of EGFP-3C^pro^ C147A had no effect (Figure 3D).

Because we also observed cleavage of TRIF in CVB3-infected cells, we determined whether 3C^pro^ was responsible for its cleavage as well. We cotransfected HEK293 cells with TRIF and either EGFP-2A^pro^, 3A, or -3C^pro^. Expression of 3C^pro^, but not 2A^pro^ or 3A, led to the cleavage of TRIF, demonstrated by a reduction in the expression of full-length TRIF and the appearance of several TRIF cleavage fragments (Figure 3E). 3C^pro^-mediated cleavage of TRIF required the cysteine protease activity of 3C^pro^ as expression of 3C^pro^ C147A did not lead to TRIF cleavage (Figure 3F). We also confirmed that 3C^pro^ was directly cleaving TRIF by incubation of Flag-TRIF purified by Flag column affinity purification from overexpressing HEK293 cells with recombinant wild-type or C147A mutant 3C^pro^. Similar to our findings with MAVS (Figure 3C), we found that only recombinant wild-type 3C^pro^ induced the appearance of TRIF cleavage fragments (Figure 3G). Note that the pattern of TRIF cleavage by *in vitro* proteolysis assay (Figure 3G) differs from our experiments with overexpressed 3C^pro^ in HEK293 cells (Figure 3E, 3F) due to the use of C-terminal CFP- versus N-terminal Flag-tagged TRIF between experiments. Taken together, our data show that 3C^pro^ directly cleaves both MAVS and TRIF.

### 3C^pro^ disrupts MAVS and TRIF type I IFN and apoptotic signaling

**Figure 4:**
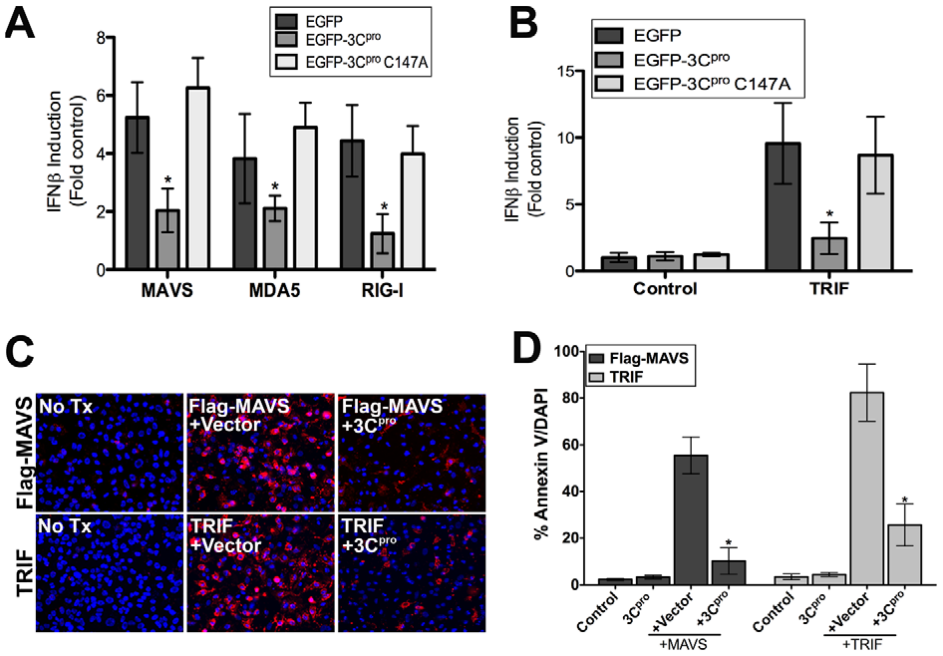
3C^pro^ abrogates MAVS and TRIF Type I IFN and apoptotic signaling. (**A**,**B**) Luciferase assay (expressed as fold IFNβ induction versus vector controls) from HEK293 cells transfected with vector, EGFP-3C^pro^ wild-type or C147A mutants, the CARDs of MAVS, MDA5, or RIG-I, and a IFNβ promoted luciferase construct. Data are shown as mean ± standard deviation. Asterisks indicate p-values ≤0.05 (**C**) Representative images of either untransfected (No Tx) U2OS cells or cells transfected with Flag-MAVS (top row) or TRIF (bottom row) and either vector alone (+Vector) or wild-type (WT) EGFP-3C^pro^. Cells were stained with Alexa Fluor 594-conjugated Annexin V 48 hrs post-transfection. Blue, DAPI-stained nuclei. (**D**) Quantification of the extent of apoptosis (shown as the percent of Annexin V positive cells/DAPI) in cells from (C). Asterisks indicate p-values ≤0.05.

To assess whether expression of 3C^pro^ abrogated MAVS-dependent signaling, we transfected HEK293 cells with wild-type or C147A EGFP-3C^pro^ or vector control, with a luciferase reporter fused to the IFNβ promoter region (p-125-Luc), and with either Flag-MAVS or the caspase activation and recruitment domains (CARDs) of MDA5 or RIG-I. Expression of the CARDs of MDA5 and RIG-I alone results in the constitutive activation of type I IFN signaling even in the absence of stimulus [31]. We found that whereas there was pronounced induction of IFNβ activity in cells expressing vector alone or EGFP-3C^pro^ C147A, expression of wild-type EGFP-3C^pro^ led to a significant reduction in promoter activity (Figure 4A).

We next determined whether 3C^pro^ attenuated TRIF-mediated signaling. TRIF is involved in the activation of IRF3 and IFNβ induction downstream of dsRNA-TLR3 engagement. While the expression of TRIF and vector control enhanced IFNβ promoter activity, expression of TRIF in combination with 3C^pro^ significantly impaired IFNβ promoter activity (Figure 4B). We found that 3C^pro^-mediated inhibition of IFNβ signaling was indeed occurring upstream of IRF3 activation as coexpression of wild-type 3C^pro^ and IRF3 had no effect on IRF3-mediated activation of IFNβ promoter activity (Supplemental Figure S4A). Furthermore, we found that expression of wild-type, but not C147A 3C^pro^ reduced IFNβ activation in response to infection with VSV (Supplemental Figure S4B).

In addition to their roles in type I IFN signaling, ectopic expression of MAVS [32] and TRIF [33] potently stimulate intrinsic apoptotic machinery to induce cell death. We found that expression of MAVS or TRIF induced pronounced apoptosis as demonstrated by enhanced Annexin V binding [which identifies the externalization of phosphatidylserine in cells undergoing apoptosis] (Figure 4C, 4D). In contrast, expression of MAVS or TRIF in the presence of 3C^pro^ potently reduced apoptosis (Figure 4C, 4D). Taken together, these data show that 3C^pro^ represses both the apoptotic and type I IFN signaling mediated by MAVS and TRIF.

### 3C^pro^ cleaves MAVS within the proline rich region

**Figure 5:**
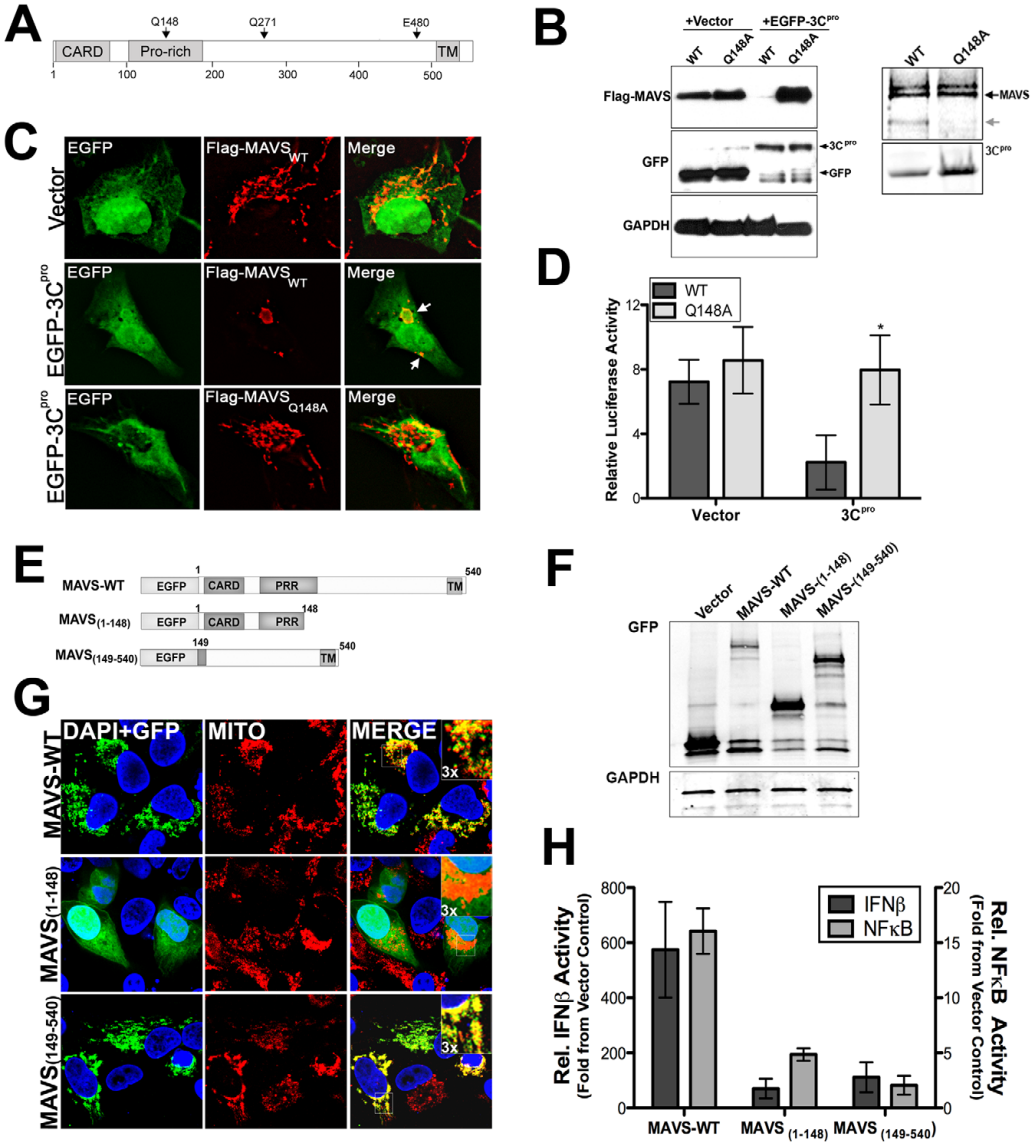
Q148 is the site of 3C^pro^-mediated cleavage of MAVS. (**A**) Schematic of MAVS showing the locations of possible 3C^pro^ cleavage sites. (**B**) Left, Immunoblot analysis for overexpressed Flag-MAVS wild-type and the Q148A mutant in HEK293 cells cotransfected with either vector or EGFP-3C^pro^. Right, recombinant wild-type SUMO-3C^pro^ (10 µg) was incubated with wild-type or Q148A column purified Flag-MAVS (0.1 µg) for 12 hrs at 37°C, fractionated by SDS-PAGE, and immunoblotted with anti-Flag monoclonal antibody (top) or commassie stained (bottom). (**C**) Immunofluorescence microscopy for Flag-MAVS wild-type (WT) of the Q148A mutant in U2OS cells co-transfected with EGFP-3C^pro^. (**D**) Luciferase assay (expressed in relative luciferase activity) from HEK293 cells transfected with wild-type or Q148A Flag-MAVS and vector control or EGFP-3C^pro^ and IFNβ promoted luciferase constructs. Data are shown as mean ± standard deviation. Asterisks indicate p-values of ≤0.05. (**E**), Schematic of EGFP-fused MAVS constructs of 3C^pro^-induced MAVS cleavage fragments. (**F**), Western blot analysis of HEK293 cells transfected with MAVS constructs depicted in (E). Lysates were immunoblotted with anti-monoclonal GFP antibody (top) or GAPDH as a loading controls (bottom). (**G**), Confocal microscopy of U2OS cells transfected with EGFP-fused MAVS 3C^pro^ cleavage fragments shown in (E). Cells were fixed and stained with anti-mitochondria monoclonal antibody (MITO) (red). Blue, DAPI stained nuclei. (**H**), Luciferase assay (expressed in relative luciferase activity) from HEK293 cells transfected with EGFP-fused MAVS 3C^pro^ cleavage fragments [from (E)] and NFκB or IFNβ promoted luciferase constructs. Data are shown as mean ± standard deviation. Asterisks indicate p-values ≤0.05.

3Cpro preferentially cleaves glutamine-glycine (Q-G) bonds in both the viral polyprotein and cellular targets, but may also exhibit proteolytic activity against glutamine-alanine (Q-A) bonds, amongst others [29]. In order to identify the residue(s) within MAVS cleaved by 3C^pro^, we constructed a panel of site directed mutants within MAVS at residues that may serve as 3C^pro^ cleavage sites (Q148, Q211, and E480) (Figure 5A). Of these mutants, only one (Q148A) was resistant to 3C^pro^-mediated cleavage in HEK293 cells and by *in vitro* protease assay (Figure 5B). Moreover, whereas wild-type Flag-MAVS was relocalized from the mitochondrial membrane upon expression of EGFP-3C^pro^, the Q148A Flag-MAVS mutant retained its mitochondrial localization (Figure 5C). The 3C^pro^ cleavage site within MAVS (Q148) is located in the proline rich region, which mediates its interaction with a number of signaling molecules including TRAF2 [4], TRAF3 [34], TRAF6 [4], RIP1 [2], and FADD [2].

We next determined whether the Q148A mutant of MAVS was resistant to 3C^pro^-mediated abatement of MAVS signaling. We found that whereas there was a pronounced reduction in IFNβ activity in cells expressing wild-type Flag-MAVS and EGFP-3C^pro^, there was no effect of EGFP-3C^pro^ expression on IFNβ signaling in cells transfected with Q148A Flag-MAVS (Figure 5D, Supplemental Figure S2D). These data show that CVB3 3C^pro^ cleaves MAVS at Q148 to suppress MAVS signaling.

### 3C^pro^-induced MAVS cleavage fragments exhibit reduced function

MAVS requires an intact CARD and localization to the mitochondrial membrane (via a C-terminal transmembrane domain) to remain functionally active [5]. Because we found that 3C^pro^ cleaved MAVS at a specific residue (Q148) within the proline rich region, we next determined whether either of the possible 3C^pro^-induced cleavage fragments of MAVS would remain active. To that end, we constructed EGFP-fused constructs expressing wild-type MAVS, the N-terminal (residues 1-148), or C-terminal (residues 149-540) fragments of MAVS that would result from 3C^pro^ cleavage (Figure 5F). We found that the N-terminal fragment of MAVS (1-148) no longer localized to the mitochondrial membrane (Figure 5G) and induced NFκB or IFNβ signaling significantly less that full-length MAVS (Figure 5H). Whereas the C-terminal fragment of MAVS (149-540) retained its mitochondrial localization (Figure 5G), it also exhibited significantly less NFκB and IFNβ activation in comparison to full-length MAVS (Figure 5H). These data indicate that 3C^pro^-mediated cleavage of MAVS likely inactivates MAVS-mediated downstream signaling by directly cleaving a residue that separates the CARD and transmembrane regions.

### 3C^pro^ localizes to the TRIF signalosome and interacts with the C-terminus of TRIF

**Figure 6:**
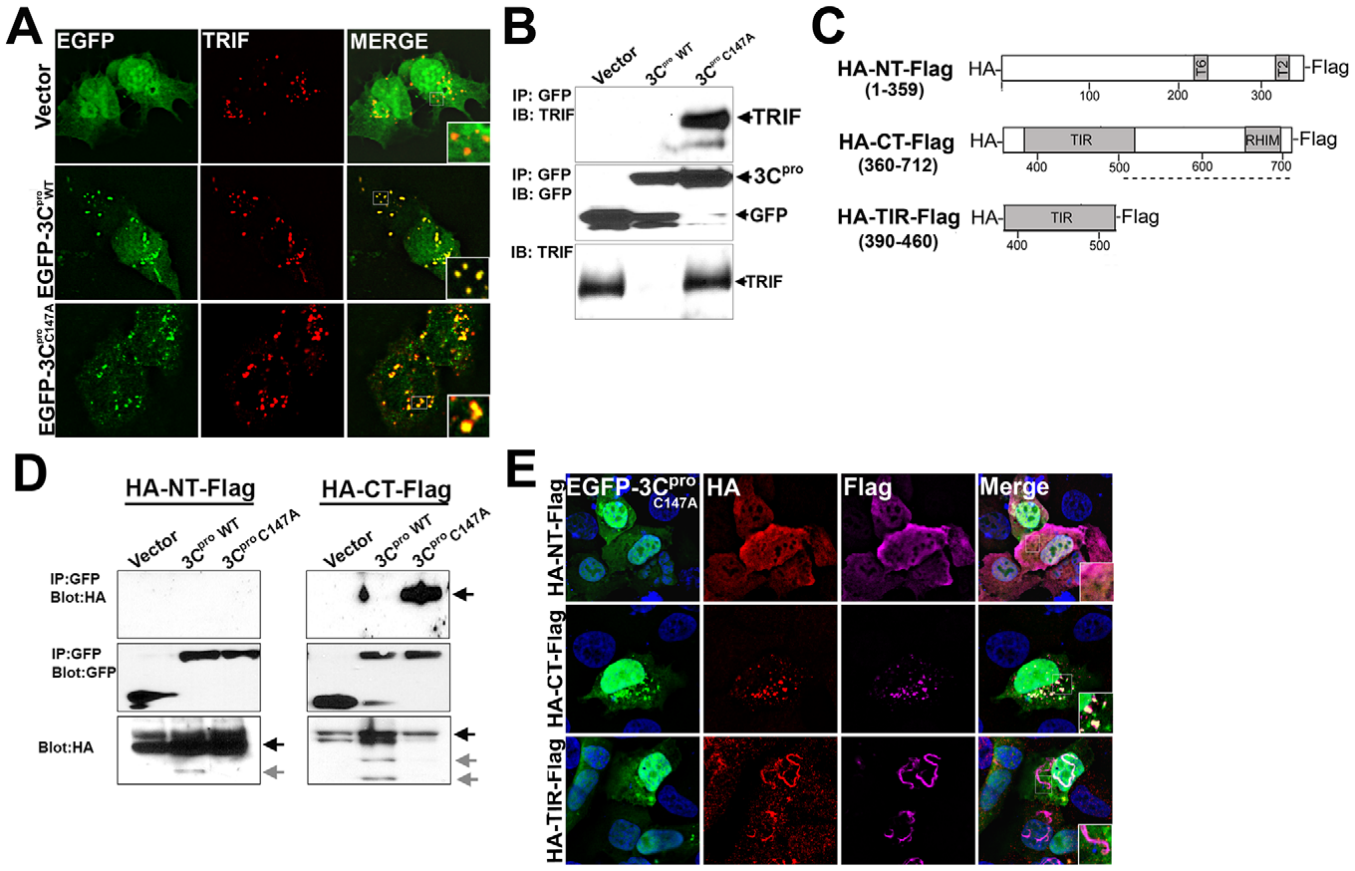
3C^pro^ localizes to TRIF signalosomes and interacts with the C-terminal domain of TRIF. (**A**) Immunofluorescence microscopy for overexpressed TRIF (in red) in U2OS cells coexpressing vector control (EGFP-C2), or wild-type or C147A mutant EGFP-3C^pro^. (**B**) HEK293 cells transfected with TRIF and vector, or wild-type or C147A mutant EGFP-3C^pro^ were lysed and subjected to immunoprecipitation with an anti-GFP monoclonal antibody. Immunoprecipitates were subjected to immunoblot analysis for TRIF and GFP. (**C**), Top, schematic of N-terminal HA-tagged and C-terminal Flag-tagged TRIF constructs. (**D**), HEK293 cells transfected with the indicated TRIF construct and either vector, wild-type or C147A mutant EGFP-3C^pro^ were lysed and subjected to immunoprecipitation with an anti-GFP monoclonal antibody. Immunoprecipitates were subjected to immunoblot analysis for HA and GFP. Arrows denote full-length (black) or cleaved (grey) TRIF. (**E**) Immunofluorescence microscopy for HA (in red), Flag (in purple), and EGFP and DAPI-stained nuclei in U2OS cells transfected with EGFP-3C^pro^ C147A and the indicated TRIF construct.

Overexpressed TRIF forms multimers and localizes to punctate cytoplasmic structures referred to as the TRIF ‘signalosome’ [35]. Downstream components of TRIF signaling localize to signalosomes as a mechanism to stimulate TRIF signaling [35], [36]. We found that EGFP-3C^pro^ and EGFP-3C^pro^ C147A were recruited to TRIF signalosomes when co-expressed with TRIF (Figure 6A). This recruitment was specific for 3C^pro^ as we did not observe the recruitment of either EGFP-2A^pro^ (not shown) or EGFP-3A (Supplemental Figure S5) to TRIF signalosomes. Although TLR3 (and presumably TRIF) can localize to endosomal membranes [37], we did not observe any colocalization of overexpressed TRIF with markers of both early and late endosomes (Supplemental Figure S6).

Because we observed the relocalization of 3C^pro^ to the signalosome complex, we next determined whether 3C^pro^ and TRIF interact within this specialized complex. HEK293 cells were transfected with TRIF and either vector (EGFP alone), EGFP-3C^pro^ wild-type, or EGFP-3C^pro^ C147A and co-immunoprecipitation studies were performed. We found that whereas EGFP-3C^pro^ C147A and TRIF co-immunoprecipitated, wild-type EGFP-3C^pro^ and TRIF did not (Figure 6B). These findings indicate that 3C^pro^ forms an interaction with TRIF that is likely abolished upon 3C^pro^-mediated cleavage.

TRIF contains a proline-rich N-terminal region, a Toll/Interleukin-1 receptor (TIR) domain, and a C-terminal region. To determine which TRIF domain is responsible for interacting with 3C^pro^ and recruiting it to the signalosome, we constructed N-terminal (NT, 1–359aa), C-terminal (CT, 360–712aa), and TIR (390–460aa) domain expression constructs of TRIF containing a HA-tag at the N-terminus and a Flag-tag at the C-terminus (Figure 6C). We then coexpressed these constructs with wild-type and C147A versions of 3C^pro^ and performed fluorescence microscopy and immunoprecipitation analysis. We found that 3C^pro^ C147A specifically interacted with the C-terminal domain of TRIF, but not the N-terminus (Figure 6D). However, the TIR domain did not mediate the interaction between TRIF and 3C^pro^ as we observed no co-immunoprecipitation between HA-TIR-Flag and 3C^pro^ (not shown).

Previous studies have shown that expression of the C-terminus of TRIF is required for the formation of the TRIF signalosome [36]. We found that expression of HA-CT-Flag was sufficient to induce the relocalization of 3C^pro^ C147A to signalosomes (Figure 6E). In contrast, 3C^pro^ C147A did not localize with either HA-NT-Flag or HA-TIR-Flag (Figure 6E). The formation of tubule-like structures induced by the expression of the TRIF TIR is consistent with previous work by others [36]. Taken together, these data indicate that 3C^pro^ interacts with the C-terminus of TRIF that is sufficient for its recruitment into the TRIF signalosome.

### 3C^pro^ cleaves the N- and C-terminal regions of TRIF

**Figure 7:**
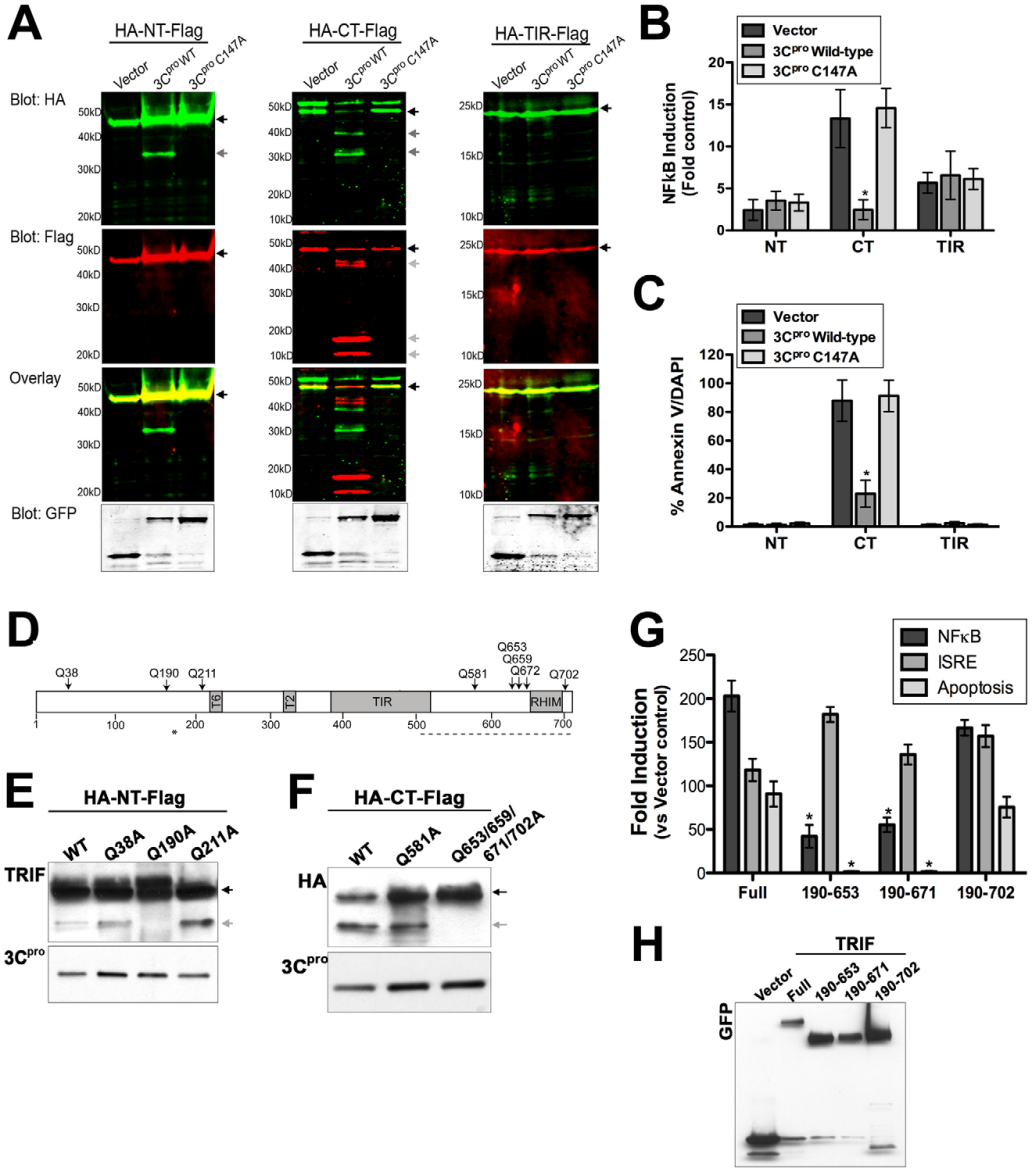
3C^pro^ cleaves the N- and C-terminal domains of TRIF at specific sites. (**A**) Dual-color immunoblot analysis using a LI-COR Odyssey infrared imaging system and antibodies specific for HA (800 nm, green) and Flag (700 nm, red) in HEK293 cells transfected with vector, EGFP-3C^pro^ wild-type or C147A and the indicated TRIF plasmids (for schematic, see Figure 6C). Black arrows denote full-length TRIF and grey arrows denote cleavage fragments. An overlay of both channels is shown below (with yellow indicating overlapping signals). (**B**) Luciferase assay (expressed in relative luciferase activity) from HEK293 cells transfected with vector, wild-type or C147A EGFP-3C^pro^, the indicated domains of TRIF, and a NFκB promoted luciferase construct. (**C**), Quantification of the extent of apoptosis (shown as the percent of Annexin V positive cells/DAPI) in HEK293 cells transfected with the indicated domains of TRIF and vector alone or wild-type of C147A mutant EGFP-3C^pro^. (**D**) Schematic of TRIF showing the locations of possible 3C^pro^ cleavage sites. (**E**,**F**) Immunoblot analysis for wild-type or 3C^pro^-resistant mutants of the N-terminal (HA-NT-Flag) (E) or C-terminal (HA-CT-Flag) (F) domains of TRIF from lysates of HEK293 cells transfected with the indicate constructs and EGFP-3C^pro^. Immunblots were performed with anti-TRIF [NT-TRIF, (E)] or anti-HA [CT-TRIF, (F)]. (**G**). HEK293 cells were transfected with EGFP-fused full-length TRIF (Full) or possible possible 3C^pro^-induced cleavage fragments of TRIF. Cells were either co-transfected NFκB and IFNβ promoted luciferase constructs and luciferase assays performed or the extent of apoptosis was measured by AnnexinV binding. Data are presented as fold-induction versus vector controls. (**H**), Lysates from cells described in (G) were harvested and immunoblotted with anti-GFP monoclonal antibody. Data in (B), (C), and (G) are shown as mean ± standard deviation. Asterisks indicate p-values ≤0.05.

We did not observe any interaction between wild-type 3C^pro^ and either full-length or C-terminal TRIF (Figure 6D and 6E) suggesting that the interaction between TRIF and 3C^pro^ is diminished following cleavage. Interestingly, we observed the appearance of cleavage fragments of both HA-NT-Flag and HA-CT-Flag when coexpressed with wild-type 3C^pro^ (Figure 6D). To further define the extent of 3C^pro^-mediated proteolysis of the N- and C-terminal regions of TRIF, we coexpressed dually HA- and Flag-tagged constructs of TRIF (described in Figure 6C) and wild-type or C147A EGFP-3C^pro^ and subjected lysates to dual-color (700 nm and 800 nm) immunoblot analysis using a LI-COR Odyssey infrared imaging system and antibodies specific for HA and Flag. This technique could therefore allow for the detection of a variety of TRIF cleavage fragments simultaneously. We found that expression of wild-type 3C^pro^ (but not the C147A mutant) induced the cleavage of both the N- and C-termini of TRIF (Figure 7A). In contrast, we observed no cleavage of the TIR domain (Figure 7A). Additionally, our data indicate that the C-terminus of TRIF is cleaved more abundantly than the N-terminus as we observed a marked decrease in the expression of full-length HA-CT-Flag and the appearance of several HA- or Flag-tag-positive cleavage products induced by 3C^pro^ overexpression (Figure 7A).

### 3C^pro^ suppresses NFκB and Apoptotic signaling and via the C-terminus of TRIF

The N- and C-terminal regions of TRIF differ in their capacities to induce type I IFN and NFκB signaling—whereas overexpression of the N-terminal region of TRIF activates both IFNβ and NFκB signaling, the C-terminal domain fails to activate IFNβ but potently induces NFκB activation [38], [39]. Moreover, the C-terminus of TRIF is sufficient to induce apoptosis [33]. The RIP homotypic interaction motif (RHIM) at the C-terminus of TRIF is essential for both NFκB and apoptotic signaling [33], [40]. Because we observed pronounced 3C^pro^-mediated cleavage of the C-terminus of TRIF (Figure 7A), we investigated whether NFκB and apoptotic signaling mediated by the C-terminus of TRIF was abolished. We found that expression of wild-type 3C^pro^ potently abrogated NFκB and apoptotic signaling induced by expression of the C-terminus of TRIF (Figure 7B, 7C). These findings are consistent with those indicating that 3C^pro^ also inhibits full-length TRIF-mediated apoptotic signaling (Figure 4C, 4D).

### 3C^pro^ cleaves specific sites in the N- and C-terminal regions of TRIF

In order to identify the residue(s) within TRIF cleaved by 3C^pro^, we constructed a panel of site directed mutants within the TRIF N- and C-terminal domains at residues that may serve as 3C^pro^ cleavage sites (Figure 7D). [We omitted any potential sites within the TRIF TIR domain as we did not observe 3C^pro^-induced cleavage of this domain (Figure 7A)]. We found that a specific residue (Q190) within the N-terminal region of TRIF was targeted by 3C^pro^ as mutagenesis of this site abolished 3C^pro^-induced cleavage (Figure 7E). Because several sites in the C-terminal domain of TRIF can serve as possible 3C^pro^ cleavage sites, and because these sites lie within close proximity to one another, we mutated these sites simultaneously. We found that simultaneous mutagenesis of four potential 3C^pro^ cleavage sites (Q653, Q659, Q671, and Q702) was sufficient to prevent 3C^pro^ cleavage (Figure 7F). These findings are consistent with our observation that the C-terminus of TRIF likely undergoes 3C^pro^ cleavage at several sites (Figure 7A).

### 3C^pro^-induced TRIF cleavage fragments are nonfunctional in NFκB and apoptotic signaling

Because the N- and C-terminal domains of TRIF function in unique capacities to induce IRF3, NFκB, and apoptotic signaling, we next explored whether possible 3C^pro^ cleavage fragments of TRIF could remain functional in these pathways. We constructed EGFP-fused full-length TRIF and various possible cleavage fragments of TRIF (encoding residues 190-653, 190-671, or 190-702). We found that all three possible 3C^pro^ TRIF cleavage fragments maintained their capacity to activate type I IFN signaling (as assessed by luciferase assays for IFN- stimulated response element (ISRE), an IRF3-dependent promoter) (Figure 7G, 7H). In contrast, two of these fragments, 190–653 and 190-671, lost their ability to activate NFκB or induce apoptotic signaling (Figure 7G, 7H). These data indicate that 3C^pro^-mediated cleavage of TRIF may primarily function to suppress TRIF-mediated NFκB and apoptotic signal propagation.

## Discussion

The host innate immune response to viral infections often involves the activation of parallel PRR pathways that converge on the induction of type I IFNs and NFκB activation. Several viruses have evolved sophisticated mechanisms to evade the host innate immune response by directly interfering with the activation and/or downstream signaling events associated with PRR signal propagation. Here we show that the 3C^pro^ cysteine protease of CVB3 targets MAVS and TRIF, two key adaptor molecules in the innate immune response as a mechanism to suppress type I IFN and apoptotic signaling. By targeting these adaptors, CVB3 has evolved a strategy to suppress antiviral signal propagation through two powerful pathways—TLR3 and RIG-I/MDA5. 3C^pro^ cleaves MAVS at a specific site within its proline-rich region (at Q148) and suppresses MAVS-mediated induction of type I IFNs and apoptosis. Moreover, 3C^pro^ targets both the N- and C-terminal domains of TRIF to abrogate its type I IFN, NFκB, and apoptotic signaling capacities. Interestingly, we found that 3C^pro^ localized to TRIF signalosomes and interacted with the C-terminal domain of TRIF. Taken together, these data highlight the strategies used by CVB3 to evade the host innate immune response.

Many viruses target molecules upstream of IFN induction as a means to escape host immunity. Similar to our findings with CVB3 3C^pro^, the 3C^pro^ of HAV directly cleaves MAVS to escape host immunity [8], but it is not known if HAV 3C^pro^ also cleaves TRIF. However, although HAV 3C^pro^ is responsible for mediating MAVS cleavage, the protease must be localized to the mitochondrial membrane via a transmembrane domain within the 3A viral protein in order to facilitate this event [8]. In contrast, CVB3 3A localizes to the ER membrane where it disrupts ER-Golgi vesicular trafficking [41], [42] and is thus not targeted to the mitochondrial membrane. Our studies indicate that in contrast to HAV, CVB3 3C^pro^ alone is sufficient to induce MAVS cleavage despite it not being localized to the mitochondrial membrane.

Although MAVS and TRIF are potent inducers of type I IFN signaling downstream of PRR activation, they have also been shown to induce apoptotic signaling–another powerful pathway used by host cells to suppress viral replication and progeny release. Enteroviruses are lytic viruses, and as such, possess no known mechanism for progeny release other than the destruction of the host cell membrane. Lytic viruses often develop efficient strategies to tightly regulate host cell death pathways in order to avoid killing the host cell prematurely (and terminating viral replication). CVB3 possesses anti-apoptotic strategies, which are mediated by the 2B and 2BC viral proteins [43], [44]. In addition, it has been shown that 3C^pro^ targets the inhibitor of κBα as a means to stimulate apoptosis and suppress viral replication [45]. Our results show that 3C^pro^ may also serve in an anti-apoptotic capacity to suppress MAVS- and TRIF-mediated apoptotic signaling as a means to tightly regulate host cell apoptotic pathways. The pro-apoptotic signaling mediated by MAVS requires its localization to the mitochondrial membrane and the presence of intact CARDs, but not the presence of an intact proline-rich region [32]. Although 3C^pro^ cleaves MAVS within the proline-rich region (Q148, Figure 5B, 5C), this cleavage both induces the relocalization of MAVS from the mitochondrial membrane (Figure 5C) and inhibits MAVS signals (Figure 5D). Furthermore, 3C^pro^ cleavage fragments of MAVS are non-functional (Figure 5H). Thus, the loss of MAVS-induced apoptosis in CVB3 3C^pro^-expressing cells is likely the result of both the relocalization of MAVS from the mitochondrial membrane and the inhibition of signaling via the CARD regions. Moreover, CVB3 3C^pro^ targets the C-terminal region of TRIF, which has been shown to induce apoptosis via direct binding to receptor interacting protein 1 (RIP1) via its RIP homotypic interaction motif (RHIM) [33]. Specifically, we found that 3C^pro^ targeted several sites within the C-terminal domain of TRIF that could effectively remove the RHIM domain, a domain of TRIF known to be critically involved in NFκB and apoptotic signaling (Figure 7D, F). In support of this, we found that 3C^pro^ cleavage fragments were deficient in NFκB activation and apoptosis (Figure 7G). Taken together, these data indicate that 3C^pro^ suppresses MAVS and TRIF-induced apoptotic signals both by their direct cleavage and by their relocalization from either the mitochondria or signalosome, respectively.

The N- and C-terminal domains of TRIF serve disparate functions in the initiation of innate immune signaling. Whereas the N-terminus of TRIF activates type I IFN induction via the phosphorylation of IRF3, the C-terminal domain activates NFκB [38], [39]. Interestingly, we found that 3C^pro^ cleaves both of these domains—likely as a mechanism to suppress global TRIF-generated signaling capacities. Upon ligand stimulation of TLR3 (or upon overexpression), activated TRIF forms signalosomes enriched in TRIF-associated signaling components including RIP1 and NFκB -activating kinase-associated protein 1 (NAP1) [35], [36]. We found that 3C^pro^ localizes to the TRIF signalosome and that expression of the C-terminal domain of TRIF is sufficient to induce this localization (Figure 6A, 6E). Moreover, we found that 3C^pro^ interacts with the C-terminal domain of TRIF (Figure 6D). However, it remains unclear whether this interaction is direct or mediated via an adaptor molecule that also localizes to the signalosome. Additionally, we found that 3C^pro^ cleavage of the TRIF C-terminal domain leads to the disruption of TRIF signalosome formation (Supplemental Figure S7), which is required for the initiation of TRIF-mediated IRF3 and NFκB activation [36]. It is thus conceivable that 3C^pro^ attenuates TRIF-dependent signaling via direct cleavage, the degradation of the signalosome complex, and inhibition of the interactions between TRIF and downstream molecules that are required to propagate TRIF-dependent signals.

Although we found that 3C^pro^ cleavage fragments of TRIF were deficient in NFκB and apoptotic signaling, they retained their capacity to induce type I IFN signaling (Figure 7G). These data may indicate that the cleavage fragments of TRIF generated by 3C^pro^ cleavage are short-lived and do not accumulate within the cell. In support of this, we failed to identify TRIF cleavage products induced by CVB3 infection endogenously (Figure 2F, 2G). Alternatively, it remains possible that 3C^pro^-mediated disruption of TRIF signaling is not involved in the suppression of type I IFN signaling, but may instead target type II IFN signaling. Previous studies in TLR3 and TRIF deficient mouse models indicate that TLR3- and TRIF-mediated IFNγ production plays an important role in CVB3 infections *in vivo* [19]. Thus, TLR3 signaling via TRIF to induce type II IFNs may function as a parallel pathway to MDA5 and/or RIG-I-mediated induction of type I IFNs. In this scenario, 3C^pro^ would suppress the downstream propagation of both type I and II IFN signaling in order to evade host immunity.

Viruses often utilize elegant strategies to attenuate innate immune signaling in order to promote their propagation. Here we show that the 3C^pro^ cysteine protease of CVB3 (and likely other enteroviruses) attenuates innate immune signaling mediated by two potent antiviral adapter molecules, MAVS and TRIF. By utilizing a variety of methods to abate MAVS and TRIF signaling, including both cleavage and retargeting from sites of signal propagation, 3C^pro^ can efficiently suppress both type I IFN and apoptotic signals aimed at clearing CVB3 infections. A better understanding of the mechanisms employed by enteroviruses to suppress host antiviral signaling could lead to the development of therapeutic interventions aimed at modulating viral pathogenesis.

## Methods

### Cells and viruses

Human embryonic kidney (HEK) 293, HeLa, and U2OS cells were cultured in DMEM-H supplemented with 10% FBS and penicillin/streptomycin. Human intestinal Caco-2 cells were cultured in MEM supplemented with 10% FBS and penicillin/streptomycin. Cells were screened for mycoplasma using a PCR-based mycoplasma test (Takara Bio USA) to prevent abnormalities in cellular morphology, transfection, and growth.

All experiments were performed with CVB3-RD, expanded as described [46]. Vesicular stomatitis virus (VSV) was kindly provided by Sara Cherry (University of Pennsylvania, Philadelphia, PA). Experiments measuring productive virus infection were performed with 0.1-1 plaque forming units (PFU)/cell for the indicated times. HeLa cells were infected with echovirus 7 and enterovirus 71 at a MOI = 0.1 for the indicated times.

Mouse infections were performed as described previously [47] and lysates kindly provided to us by Jeffrey M. Bergelson, Children's Hospital of Philadelphia.

### Transfections

Plasmid transfections were performed using FuGENE 6 according to the manufacturer's protocol (Roche Applied Science). Following transfection, cells were plated as described above and used 48–72 hrs later.

### Immunofluorescence microscopy

Cells cultured in collagen-coated chamber slides (LabTek, Nunc) were washed and fixed with either 4% paraformaldehyde or with ice-cold methanol. Cells were then permeabilized with 0.1% Triton X-100 in phosphate buffered saline (PBS) and incubated with the indicated primary antibodies for 1 hr at room temperature (RT). Following washing, cells were incubated with secondary antibodies for 30 min at room temperature, washed, and mounted with Vectashield (Vector Laboratories) containing 4′,6-diamidino-2-phenylindole (DAPI). For detection of apoptosis, cells were washed in cold PBS and incubated with Alexa-Fluor-488 conjugated-annexin V and propidium iodide for 15 min at room temperature. Cells were then washed, fixed in 4% paraformaldehyde, and images captured as described below. Images were captured using an Olympus IX81 inverted microscope equipped with a motorized *Z*-axis drive. Images were generated by multiple-section stacking (0.2 mm stacks) and deconvolved using a calculated point-spread function (Slidebook 5.0). Confocal microscopy was performed with a FV1000 confocal laser scanning microscope (Olympus).

### Antibodies

Rabbit polyclonal and mouse monoclonal antibodies directed against GFP (FL, B-2), GAPDH, HA (Y-11, F-7) and IRF3 (FL-425) were purchased from Santa Cruz Biotechnology. Mouse monoclonal anti-Flag (M2) was purchased from Sigma. Rabbit polyclonal antibodies to TRIF and MAVS (human and rodent specific) were purchased from Cell Signaling Technologies or Bethyl Laboratories, respectively. Mouse anti-enterovirus VP1 (Ncl-Entero) was obtained from Novocastra Laboratories (Newcastle upon Tyne, United Kingdom). Mitochondria antibody [MTC02] was purchased from Abcam. Mouse anti-enterovirus 71 antibody was purchased from Millipore. Alexa Fluor-conjugated secondary antibodies were purchased from Invitrogen.

### Plasmids

Flag-MDA5, Flag-MAVS, and Flag-TRIF plasmids were kindly provided by Tianyi Wang (University of Pittsburgh). pUNO2-hTRIF was purchased from Invivogen. EGFP-2A^pro^ 2B, 2C, 3A and -3C^pro^ were constructed by amplification from CVB33 cDNA (kindly provided by Jeffrey Bergelson, Children's Hospital of Philadelphia) and cloned into the NT-GFP TOPO fusion vector (Invitrogen) following PCR amplification. EGFP-fusion constructs expressing cleavage fragments of MAVS and TRIF were generated by PCR amplification from Flag-MAVS or pUNO2-TRIF and cloned into the NT-GFP TOPO fusion vector (Invitrogen). CFP-TRIF was purchased from Addgene (plasmid 13644). Dual HA- and Flag-tagged TRIF constructs were generated by amplification of TRIF cDNA with primers encoding a N-terminal HA or C-terminal Flag tags and cloned into the XhoI and EcoRI sites of pcDNA3.1(+). Mutagenesis of 3C^pro^, MAVS and TRIF constructs were performed using Quickchange mutagenesis kit following the manufacturer's protocol (Stratagene). Primer sequences are available upon request.

### Immunoblots and immunoprecipitations

Cell lysates were prepared with RIPA buffer (50 mM Tris-HCl [pH 7.4]; 1% NP-40; 0.25% sodium deoxycholate; 150 mM NaCl; 1 mM EDTA; 1 mM phenylmethanesulfonyl fluoride; 1 mg/ml aprotinin, leupeptin, and pepstatin; 1 mM sodium orthovanadate), and insoluble material was cleared by centrifugation at 700×g for 5 min at 4°C. Lysates (30-50 µg) were loaded onto 4–20% Tris-HCl gels (Bio-Rad, Hercules, CA) and transferred to polyvinylidene difluoride membranes. Membranes were blocked in 5% nonfat dry milk or 3% bovine serum albumin, probed with the indicated antibodies, and developed with horseradish peroxidase-conjugated secondary antibodies (Santa Cruz Biotechology), and SuperSignal West Pico or West Dura chemiluminescent substrates (Pierce Biotechnology).

Immunoblots in isolated mouse hearts and dually HA- and Flag-tagged TRIF constructs were conducted using an Odyssey Infrared Imaging System (LI-COR Biosciences). Tissue homogenized in lysis buffer (100 µg) or whole-cell lysates from transfected HEK293 cells (30 µg) were loaded onto 4–20% Tris-HCl gels, separated electrophoretically, and transferred to nitrocellulose membranes. Membranes were blocked in Odyssey Blocking buffer and then incubated with the appropriate antibodies overnight at 4°C in Odyssey Blocking buffer. Following washing, membranes were incubated with anti-rabbit or anti-mouse antibodies conjugated to IRDye 680 or 800CW and visualized with the Odyssey Infrared Imaging System according to the manufacturer's instructions.

For immunoprecipitations, HEK293 cells transiently transfected with the indicated plasmids were lysed with EBC buffer (50 mM Tris [pH 8.0], 120 mM NaCl, 0.5% Nonidet P-40, 1 mm phenylmethylsulfonyl fluoride, 0.5 µg/ml leupeptin, and 0.5 µg/ml pepstatin). Insoluble material was cleared by centrifugation. Lysates were incubated with the indicated antibodies in EBC buffer for 1 hr at 4°C followed by the addition of Sepharose G beads for an additional 1 hr at 4°C. After centrifugation, the beads were washed in NETN buffer (150 mm NaCl, 1 mm EDTA, 50 mm Tris-HCl (pH 7.8), 1% Nonidet P-40, 1 mm phenylmethylsulfonyl fluoride, 0.5 µg/ml leupeptin, and 0.5 µg/ml pepstatin), then heated at 95°C for 10 min in Laemmli sample buffer. Following a brief centrifugation, the supernatant was immunblotted with the indicated antibodies as described above.

### Expression and purification of recombinant proteins

Bacterial expression vectors encoding wild-type or C147A 3C^pro^ were constructed in pET-SUMO (which encodes a linear fusion consisting of an N-terminal 6xHis tag for affinity purification followed by SUMO) following PCR amplification according to the manufacturers protocol (Invitrogen). pET-SUMO-3C^pro^ constructs were expressed in bacteria and purified by metal chelation resin columns (Qiagen). The resulting 6xHis-SUMO 3C^pro^ fusion proteins were treated with SUMO protease to create untagged proteins as per the manufacturer's instructions (Invitrogen) and then dialyzed.

For purification of MAVS and TRIF, 10 cm dishes of HEK2393 cells were transfected with Flag-MAVS or Flag-TRIF and lysed 48 hrs post-transfection. Lysates were purified over anti-Flag affinity gel columns, washed several times, and protein eluted by competition with five washes of 3x Flag peptide using the Flag M purification kit for mammalian expression systems (Sigma-Aldrich). Eluted protein was quantified by BCA protein assay and verified for purity by SDS-PAGE and immunoblot analysis.

### Reporter gene assay

Activation of the NFκB and IFNβ promoter was measured by reporter assay. Cells were transfected in 24-well plates with p-125 luc (IFNβ) or NFκB reporter plasmid together with the indicated plasmids. Luciferase activity was measured by the Dual-Luciferase assay kit (Promega). All experiments were performed in triplicate and conducted a minimum of three times.

### ELISA

To measure IFNβ production, the indicated cells were infected with either CVB or VSV and samples of culture supernatant removed at the indicated times. IFNβ levels in culture supernatant were determined by IFNβ ELISA according to the manufacturer's instructions (PBL Biomedical Laboratories).

### Isolation of nuclear extracts

Nuclear extracts were prepared from HEK293 cells after infection with CVB3 for 12 hr. Cells were washed in ice-cold PBS and isolated by incubation in 10 mM EDTA for 10 min. Cells were pelleted at 1000×g for 5 min, washed in ice-cold PBS, and incubated with buffer A (10 mM HEPES [pH 7.9], 1.5 mM MgCl2, 10 mM KCl, 0.5 mM DTT, 0.5 mM PMSF, and 0.1% NP-40). The pellets were then resuspended in buffer B (20 mM HEPES [pH 7.9], 25% glycerol, 0.42 M NaCl, 1.5 mM MgCl2, 0.2 mM EDTA, 0.5 mM DTT, 0.5 mM PMSF, 5-µg/ml leupeptin, 5-µg/ml pepstatin, 5-µg/ml aprotinin). Samples were incubated on ice for 15 min before being centrifuged at 10,000×g. Nuclear extract supernatants were diluted with buffer C (20 mM HEPES [pH 7.9], 20% glycerol, 0.2 mM EDTA, 50 mM KCl, 0.5 mM DTT, 0.5 mM PMSF).

### Statistical analysis

Data are presented as mean ± standard deviation. One-way analysis of variance (ANOVA) and Bonferroni's correction for multiple comparisons were used to determine statistical significance (p<0.05).

### Gene IDs

(numbers were taken from GenBank at Pubmed): mitochondrial antiviral signaling protein (MAVS) 57506; Toll/IL-1 receptor domain-containing adaptor inducing interferon-beta (TRIF) 148022, toll-like receptor 3 (TLR3) 7098; Retinoic acid-inducible gene-I (RIG-I) 23586; melanoma-differentiation-associated gene 5 (MDA5) 64135; interferon regulatory factor 3 (IRF3) 3661.

## Supporting Information

Figure S1 CVB infection is sensitive to type I interferons. (A) Western blot analysis for VP1 in HeLa cells pretreated with medium alone (Con) or medium containing 100 U or 1000 U of purified IFNβ for 24 hrs and then infected with CVB (1PFU/cell) for 10 hrs. (B) As a control, similar studies were performed with VSV. Western blot analysis for VSV-G in HeLa cells pretreated with medium alone (Con) or medium containing 100 U or 1000 U of purified IFNβ for 24 hrs and then infected with VSV (5PFU/cell) for 10 hrs.
(0.13 MB TIF)

Figure S2 CVB, but not VSV, infection induces MAVS cleavage. (A) Western blot analysis for MAVS in lysates from HeLa cells infected with CVB for 10 hrs in the absence (NoI) or presence of Z-VAD-FMK (zVAD) or MG132. (B) Western blot analysis for MAVS in lysates from HEK293 cells infected with CVB or VSV for 12 hrs. (C),HEK293 cells with transfected with D429E Flag-MAVS (1 µg in no virus (NoV) controls or 2 µg in CVB-infeceted culures) and then infected with CVB (1PFU/cell for 12 hrs) 48 hrs following transfection. Lysates were harvested and immunoblotted for Flag, VP1, or GAPDH (as a loading control). (D), Lysates from Figure 5D were immunoblotted for Flag and GFP.
(0.36 MB TIF)

Figure S3 MAVS and TRIF are cleaved by other enteroviruses and are absent from the hearts of CVB-infected mice. (A) Immunoblot analysis for MAVS and TRIF in HeLa cells infected with echovirus 7 (E7) or enterovirus 71 (EV71) for the indicated times (0.1 PFU/cell). (B) Hearts of three mice infected by intraperitoneal injection with CVB for 7 days were removed, homogenized, and lysed. Lysates were subjected to immunblot analysis for MAVS and TRIF using an Odyssey Infrared Imaging System (immunoblots are shown as grey scale images).
(0.17 MB TIF)

Figure S4 3Cpro acts upstream of IRF3 and attenuates VSV-induced IFNβ activation. (A), HEK293 cells were transfected with an IFNβ-luciferase construct and IRF3 either with or without 3Cpro. Lysates were harvested 48 hrs post-transfection and luciferase activity measured. (B), HEK293 cells were transfected with an IFNβ-luciferase construct and either vector control, or wild-type of C147A 3Cpro. 48 hrs post-transfections, cells were infected with VSV for 12 hrs, lysates collected and and luciferase activity measured.
(0.21 MB TIF)

Figure S5 CVB 3A does not localize to the TRIF signalosome. Immunofluoescence microscopy of U2OS cells transfected with EGFP-3A and TRIF (red).
(0.25 MB TIF)

Figure S6 TRIF does not localize to endosomes. U2OS cells transfected with TRIF were stained for early endosome antigen-1 (EEA1), the lysosomal marker LAMP2, or Alexa Fluor 488-conjugated transferrin (TRANS).
(1.72 MB TIF)

Figure S7 3Cpro inhibits signalsome formation. Immunofluoescence microscopy of EGFP-3Cpro wild-type and HA-CT-Flag (HA, red) in transfected U2OS cells.
(0.38 MB TIF)
