# Supplementary figures and images for: The Coxsackievirus B 3Cpro Protease Cleaves MAVS and TRIF to Attenuate Host Type I Interferon and Apoptotic Signaling
Source: PLoS Pathog. 2011 Mar 10;7(3):e1001311. doi: 10.1371/journal.ppat.1001311 (PMC3059221; doi:10.1371/journal.ppat.1001311)

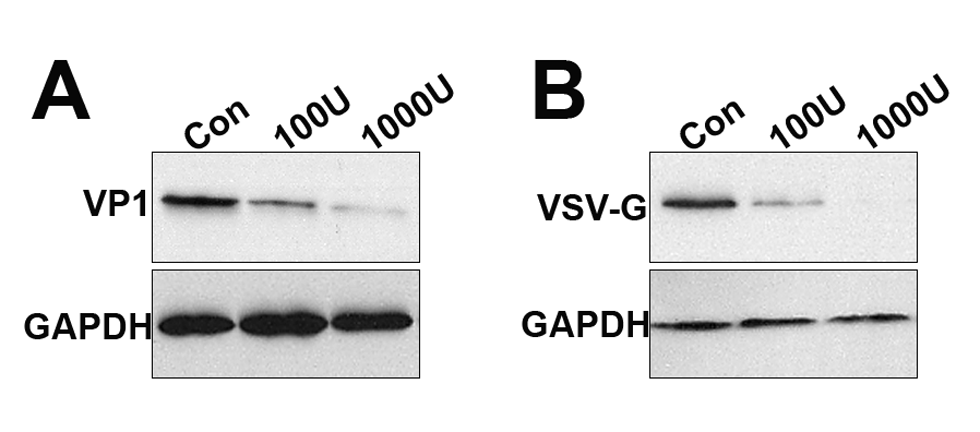

Supplement: Figure S1 — CVB infection is sensitive to type I interferons. (A) Western blot analysis for VP1 in HeLa cells pretreated with medium alone (Con) or medium containing 100 U or 1000 U of purified IFNβ for 24 hrs and then infected with CVB (1PFU/cell) for 10 hrs. (B) As a control, similar studies were performed with VSV. Western blot analysis for VSV-G in HeLa cells pretreated with medium alone (Con) or medium containing 100 U or 1000 U of purified IFNβ for 24 hrs and then infected with VSV (5PFU/cell) for 10 hrs. (0.13 MB TIF) [file ppat.1001311.s001.tif]

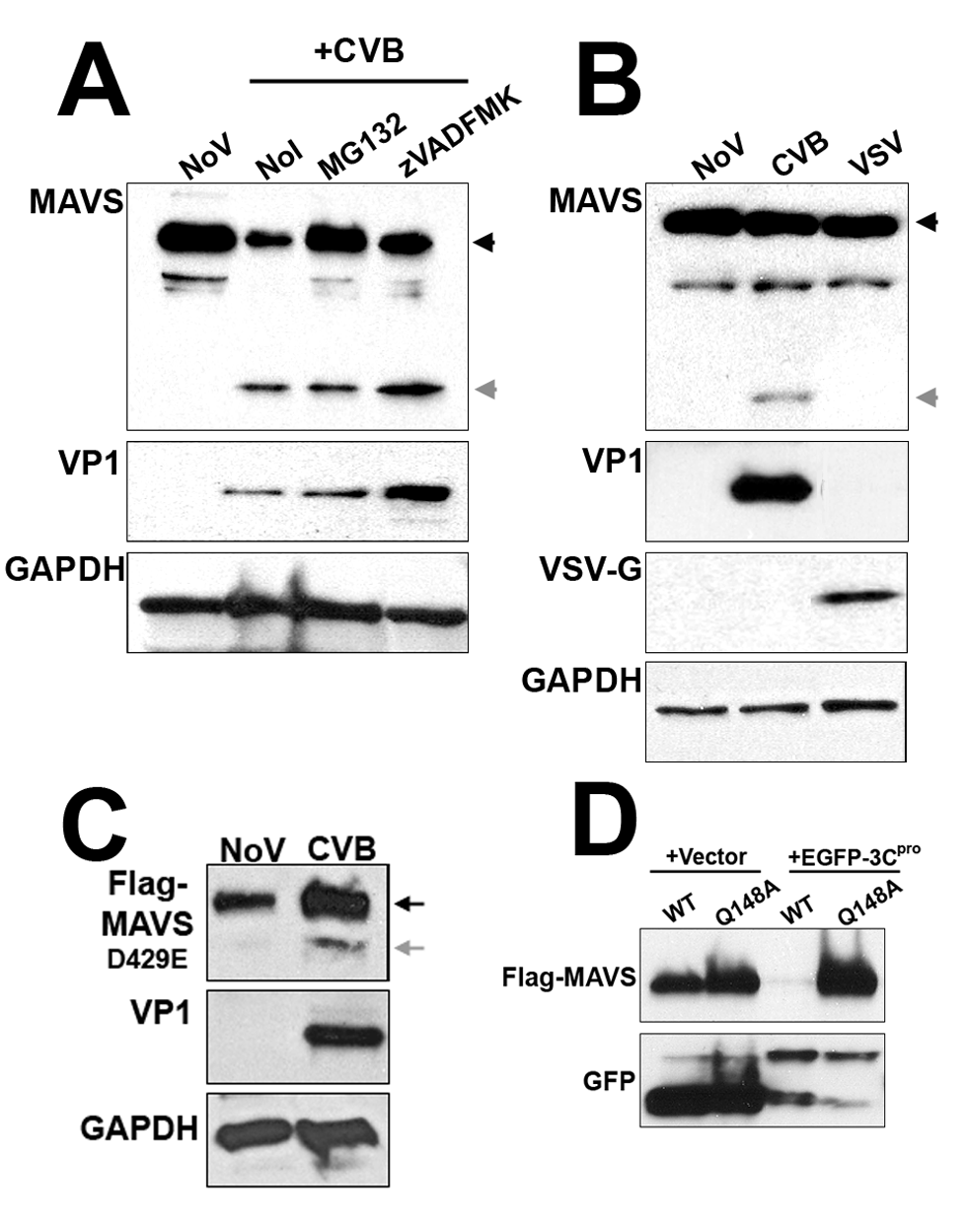

Supplement: Figure S2 — CVB, but not VSV, infection induces MAVS cleavage. (A) Western blot analysis for MAVS in lysates from HeLa cells infected with CVB for 10 hrs in the absence (NoI) or presence of Z-VAD-FMK (zVAD) or MG132. (B) Western blot analysis for MAVS in lysates from HEK293 cells infected with CVB or VSV for 12 hrs. (C),HEK293 cells with transfected with D429E Flag-MAVS (1 µg in no virus (NoV) controls or 2 µg in CVB-infeceted culures) and then infected with CVB (1PFU/cell for 12 hrs) 48 hrs following transfection. Lysates were harvested and immunoblotted for Flag, VP1, or GAPDH (as a loading control). (D), Lysates from Figure 5D were immunoblotted for Flag and GFP. (0.36 MB TIF) [file ppat.1001311.s002.tif]

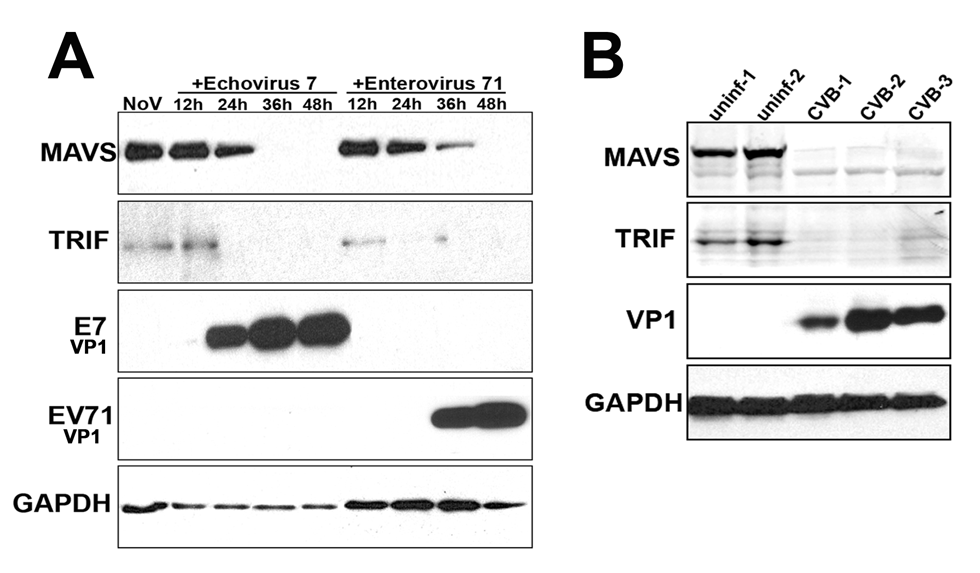

Supplement: Figure S3 — MAVS and TRIF are cleaved by other enteroviruses and are absent from the hearts of CVB-infected mice. (A) Immunoblot analysis for MAVS and TRIF in HeLa cells infected with echovirus 7 (E7) or enterovirus 71 (EV71) for the indicated times (0.1 PFU/cell). (B) Hearts of three mice infected by intraperitoneal injection with CVB for 7 days were removed, homogenized, and lysed. Lysates were subjected to immunblot analysis for MAVS and TRIF using an Odyssey Infrared Imaging System (immunoblots are shown as grey scale images). (0.17 MB TIF) [file ppat.1001311.s003.tif]

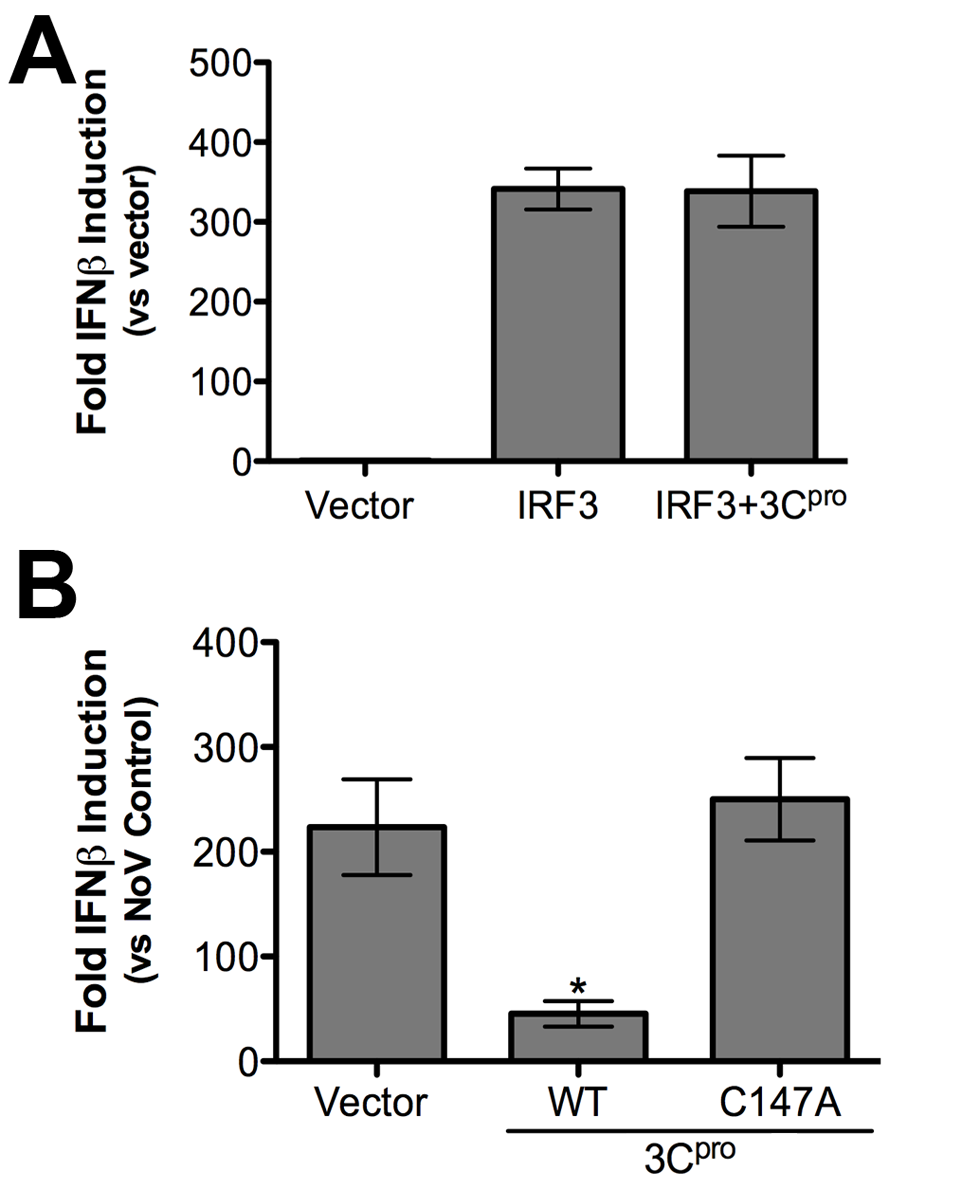

Supplement: Figure S4 — 3Cpro acts upstream of IRF3 and attenuates VSV-induced IFNβ activation. (A), HEK293 cells were transfected with an IFNβ-luciferase construct and IRF3 either with or without 3Cpro. Lysates were harvested 48 hrs post-transfection and luciferase activity measured. (B), HEK293 cells were transfected with an IFNβ-luciferase construct and either vector control, or wild-type of C147A 3Cpro. 48 hrs post-transfections, cells were infected with VSV for 12 hrs, lysates collected and and luciferase activity measured. (0.21 MB TIF) [file ppat.1001311.s004.tif]

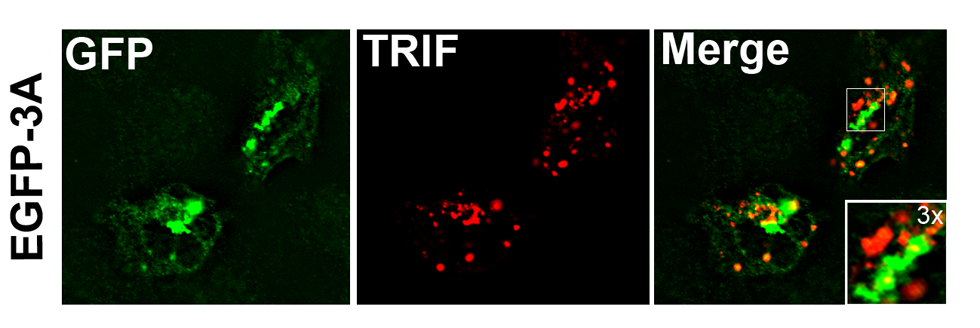

Supplement: Figure S5 — CVB 3A does not localize to the TRIF signalosome. Immunofluoescence microscopy of U2OS cells transfected with EGFP-3A and TRIF (red). (0.25 MB TIF) [file ppat.1001311.s005.tif]

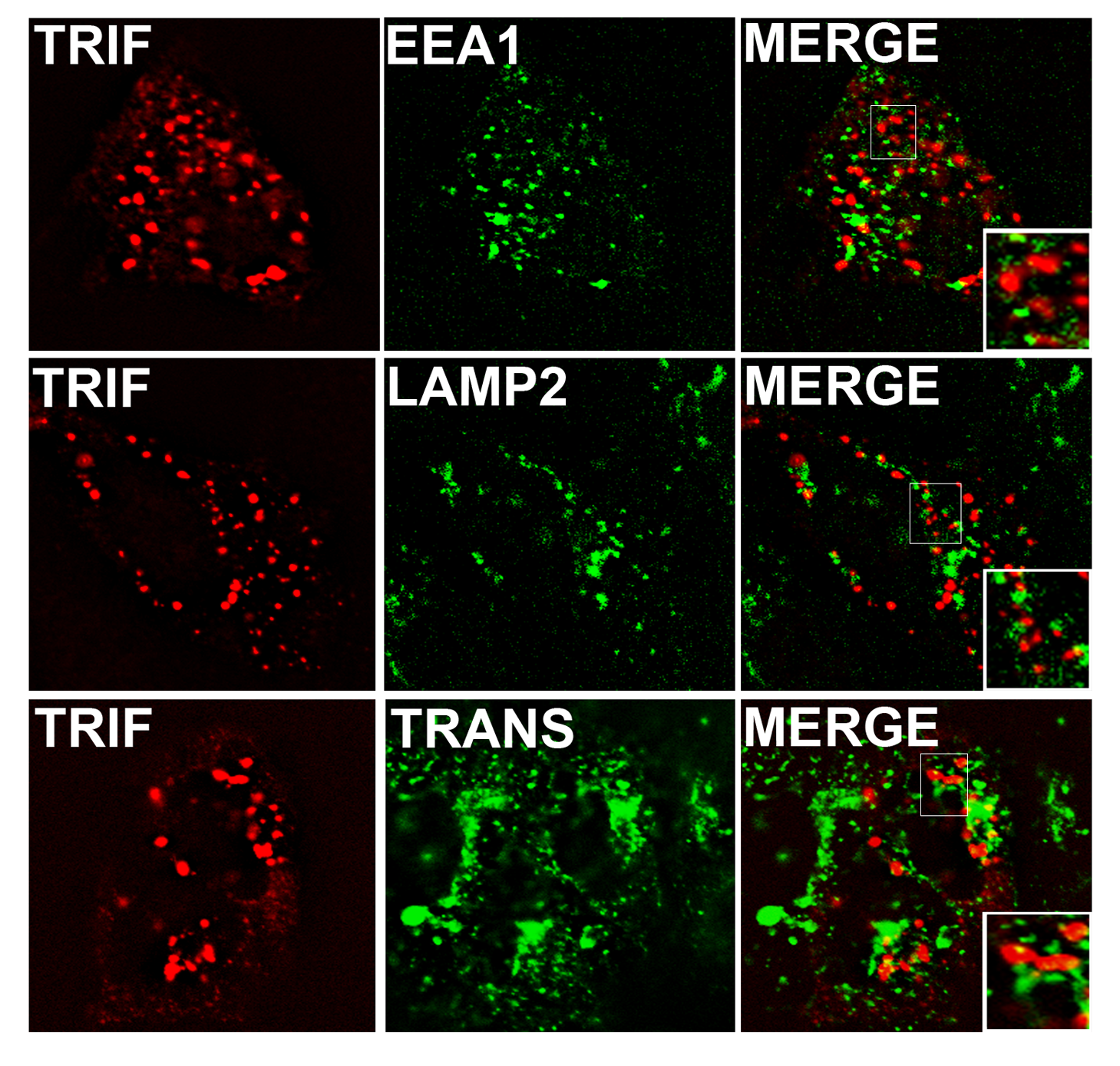

Supplement: Figure S6 — TRIF does not localize to endosomes. U2OS cells transfected with TRIF were stained for early endosome antigen-1 (EEA1), the lysosomal marker LAMP2, or Alexa Fluor 488-conjugated transferrin (TRANS). (1.72 MB TIF) [file ppat.1001311.s006.tif]

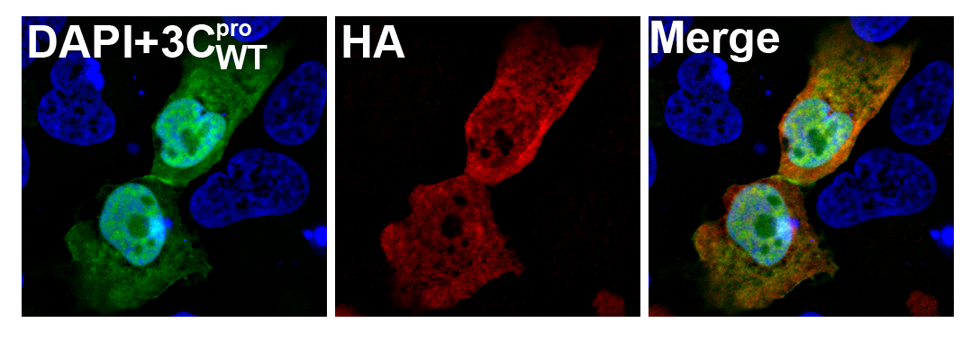

Supplement: Figure S7 — 3Cpro inhibits signalsome formation. Immunofluoescence microscopy of EGFP-3Cpro wild-type and HA-CT-Flag (HA, red) in transfected U2OS cells. (0.38 MB TIF) [file ppat.1001311.s007.tif]
